# Proof of concept: liver splitting during normothermic machine perfusion

**DOI:** 10.1093/jscr/rjx218

**Published:** 2018-03-28

**Authors:** Barney T F Stephenson, Glenn K Bonney, Richard W Laing, Ricky H Bhogal, Francesca Marcon, Desley A H Neil, M Thamara P R Perera, Simon C Afford, Hynek Mergental, Darius F Mirza

**Affiliations:** 1 NIHR Birmingham Liver Biomedical Research Unit and Centre for Liver Research, University of Birmingham, Edgbaston, Birmingham B15 2TT, UK; 2 The Liver Unit, Queen Elizabeth Hospital, University Hospitals Birmingham NHS Foundation Trust, Birmingham B15 2GW, UK

## Abstract

**Introduction:**

Despite utilizing extended criteria donors, there remains a shortage of livers for transplantation. No data exists on splitting donor livers with concurrent NMP-L.

**Methods:**

A liver recovered from a donor after circulatory death was subjected to NMP-L using a red cell based fluid. During NMP-L, a ‘classical’ left lateral + right trisegmentectomy split was performed using an integrated bipolar/ultrasonic device. After splitting, blood flow was confirmed using Doppler ultrasound in each lobe.

**Results:**

Prior to splitting, flow rates were maintained physiologically. Lactate decreased from 13.9 to 3.0 mmol/L. Lactate before and after splitting were similar in the hepatic arteries, portal veins and IVC. Doppler ultrasound demonstrated arterial and venous waveforms in both lobes after splitting.

**Conclusions:**

‘Classical’ liver splitting during NMP-L is feasible, maintaining viability of both lobes. Establishing this procedure may attenuate cold ischaemic injury, allow pre-implantation monitoring of both grafts and facilitate logistics of transplanting two grafts.

## INTRODUCTION

Each year 15% of patients on the UK liver transplant waiting list either die or been removed [1]. One strategy to increase organ availability is splitting, performed either *in situ* or *ex situ*. The most common approach is the ‘classical’ left lateral + right trisegmentectomy split. The less frequently performed full left-right split can provide livers for two adults but this is technically complex and questions remain over vascular and biliary outcomes [2].

Normothermic machine perfusion of the liver (NMP-L) was developed to attenuate IRI and improve organ utilization: early data validated by transplantation [3, 4] are encouraging. Here, we report viability testing and resuscitation of a donor liver followed by splitting with concurrent NMP-L.

## CASE REPORT

### Donor

A 69-year-old female DCD liver, BMI 34.3 kg/m^2^, DRI 3.053, with a hypoxic brain injury and a 7-day ITU admission, was initially accepted for transplantation. Withdrawal of life-sustaining treatment was conducted according to UK standard practice and 34 min later dual arterial and porto-venous perfusion commenced with 4°C UW solution, using the super-rapid technique. The liver was mildly steatotic with an iatrogenic 2.5 cm left lobe laceration and atherosclerotic arteries.

CIT was 9 h 43 min. It was subsequently declined for transplantation and subjected to NMP-L. The donor consented to research and NMP-L was approved by London-Surrey Borders National Research Ethics Service committee (13/LO/1928).

### NMP-L

The liver was prepared for modified piggyback transplantation. NMP-L commenced with a packed red cell (PRC) based fluid at 37°C using the Liver Assist device (Organ Assist, The Netherlands) via hepatic artery and portal vein cannulae. Oxygenated pulsatile flow (pressure 50 mmHg) and non-pulsatile flow (pressure 10 mmHg) perfused the liver via hepatic artery and portal vein, respectively, before recirculating via the open circuit reservoir.

During NMP-L, flow parameters, blood gases and bile production were assessed every 30 min. Homogenous perfusion, stable flow parameters, lactate < 2.0 mmol/L and evidence of bile production after 2 h fulfilled criteria for viability.

### Splitting

**Figure 1:**
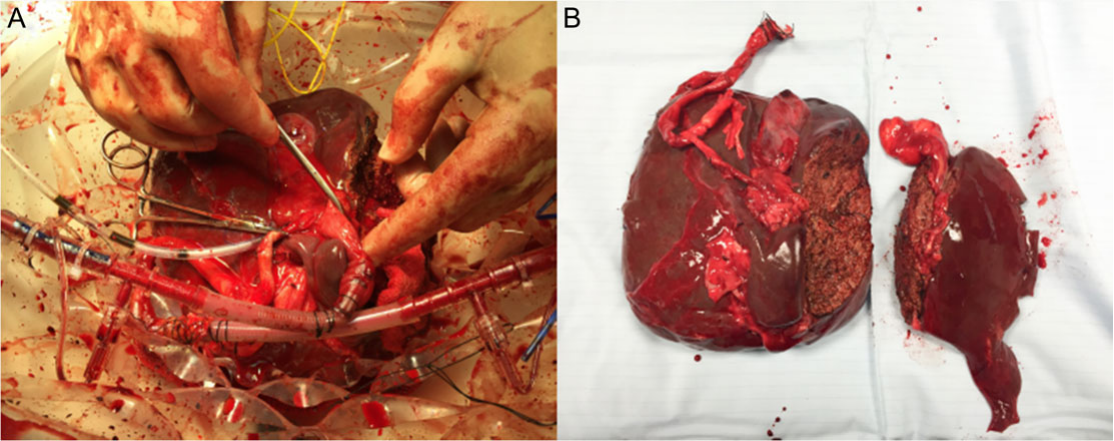
Splitting of DCD liver with concurrent NMP-L. (**A**) Appearance of the right lobe of liver after parenchymal transection whilst continuing NMP-L. (**B**) Appearance of liver lobes on completion of splitting and NMP-L.

‘Classical’ left lateral + right trisegmentectomy split was performed with an integrated bipolar/ultrasonic device (Thunderbeat, Olympus, UK) for dissection with simultaneous ligation. Splitting was conducted in the reservoir with concurrent NMP-L throughout (Fig. 1A and B, and video), maintaining inflow and outflow for both ‘grafts’.

Flow parameters and perfusate analysis were recorded throughout splitting from left and right hepatic arteries, portal veins and IVC. Post-procedure blood flow was confirmed using Doppler ultrasound (CX50 CompactXtreme, Philips, The Netherlands), in each lobe.

### Histology

Specimens taken 3 hourly and on completion of NMP-L from each lobe were examined by H&E and PAS staining. Blinded assessment was performed by a pathologist.

## RESULTS

### Pre-splitting

**Figure 2:**
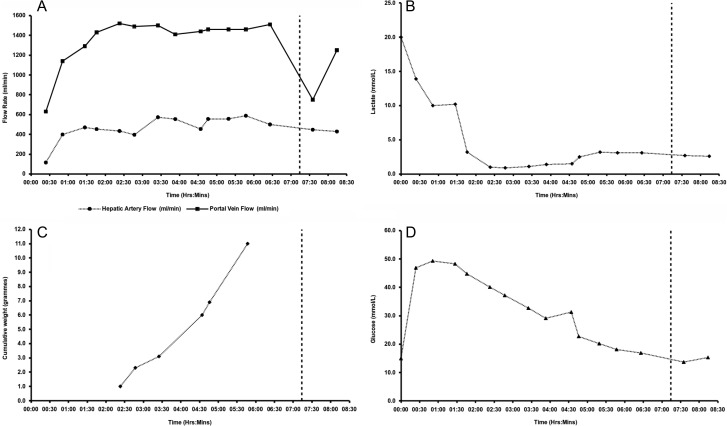
Characteristics of the liver subjected to NMP-L prior to splitting. (**A**) Hepatic arterial and portal venous flow rates; (**B**) lactate concentrations; (**C**) cumulative bile production; and (D) glucose concentrations.

The liver weighed 1650 g. Duration of pre-splitting NMP-L was 6 h 28 min. Commencing NMP-L, arterial and portal venous flow rates were 116 and 630 mL/min, respectively, reaching 573 and 1500 mL/min after 3 h (Fig. 2A): physiological pressures were maintained. Vascular resistance remained low. Initial temperature was 26°C, reaching 36°C after 47 min. Prior to NMP-L, lactate concentration was > 20.0 mmol/L and after 2 h lactate was 1.0 mmol/L (Fig. 2B), with 11 g of bile being excreted (Fig. 2C).

### Parenchymal transection

Duration of splitting with concurrent NMP-L was 71 min (total duration 8 h 22 min). Haemostasis was maintained, no additional PRC were required, suction devices were not used.

After parenchymal splitting and maintaining differential pressures (median 54 [54–55] mmHg), total hepatic arterial flow remained just below pre-hilar rates (median 429 [424–450] mL/min). Total portal venous flow rate increased to near pre-splitting rates (median 1250 [1230–60] mL/min) whilst maintaining physiological pressures (median 9 [8–10] mmHg).

**Figure 3:**
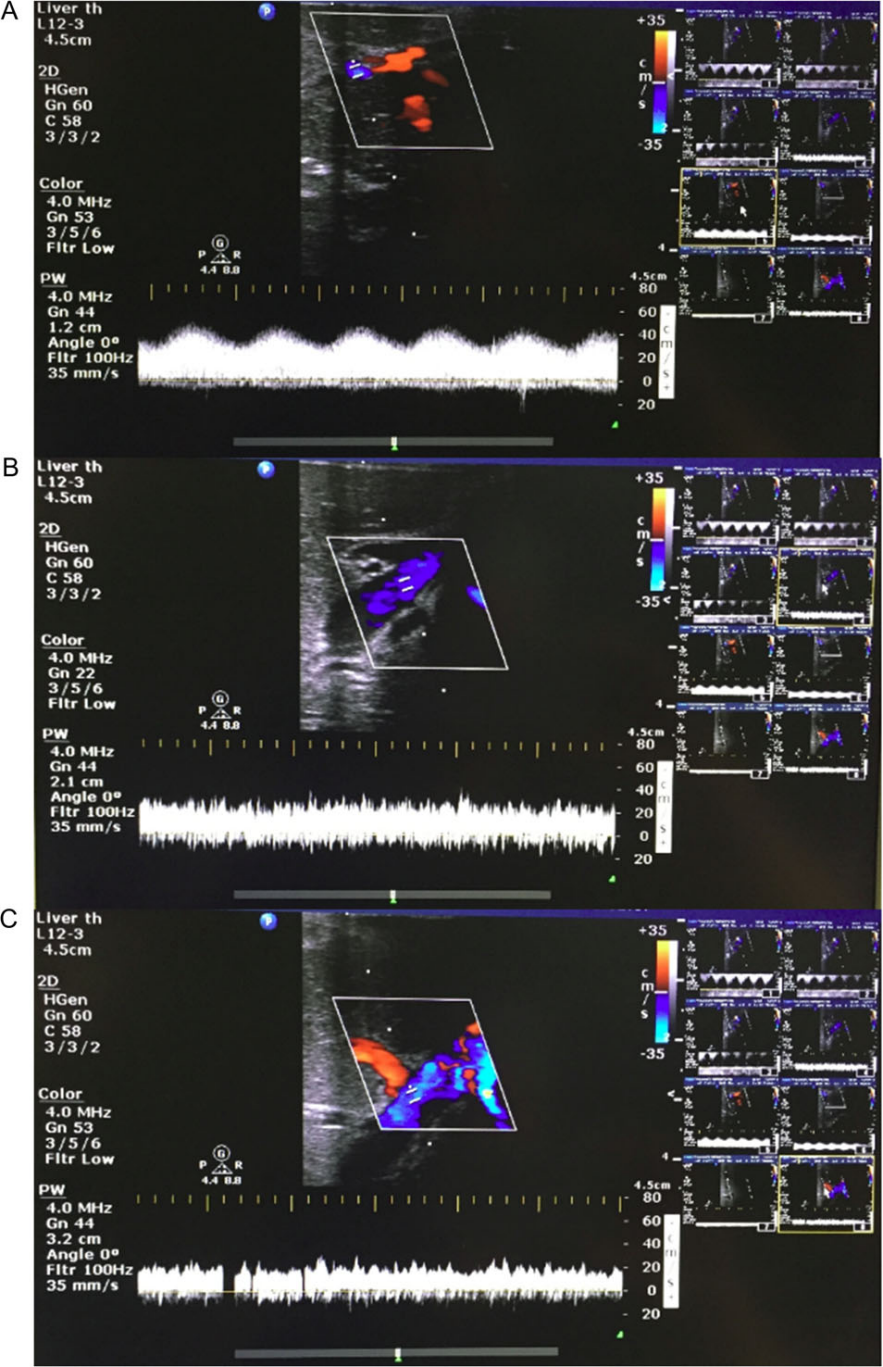
Representative Doppler ultrasound images of parenchyma from liver split with concurrent NMP-L. (**A**) Hepatic arteriolar waveform; (**B**) portal venous waveform; and (**C**) hepatic venous waveform.

Post-hilar phase lactates were similar in both hepatic arteries, portal veins and IVC (Table 1). Post-parenchymal phase lactate was unchanged. IVC Lactate increased by 0.3 mmol/L compared to the pre-hilar phase. Bile production continued throughout. Doppler ultrasound demonstrated expected hepatic arterial, portal venous and IVC waveforms in both lobes after splitting (Fig. 3).

**Table 1:** Table of flow rates and blood gas results prior to, during and after completion of splitting with concurrent NMP-L.

	Pre-parenchymal, post-hilar dissection					Post-parenchymal Transection				
	Left		Right		IVC	Left		Right		IVC
	HA	PV	HA	PV		HA	PV	HA	PV	
pH	7.439	7.432	7.442	7.448	7.387	7.416	7.405	7.439	7.403	7.369
pCO_2_ (kPa)	4.04	4.22	4.10	4.06	4.83	3.96	4.29	3.74	4.32	4.93
pO_2_ (kPa)	10.07	6.80	9.47	7.02	4.67	7.94	5.83	10.21	6.28	3.48
BE (mmol/L)	−3.6	−3.2	−3.1	−2.9	−3.3	−5.2	−4.5	−4.9	−4.4	−4.0
HCO_3_^−^ (mmol/L)	20.0	20.6	20.5	20.6	21.3	18.7	19.7	18.6	19.8	20.9
tHb (g/L)	76.5	78.2	76.7	77.5	81.0	78.1	78.4	79.5	79.0	76.1
Hct (%)	17.2	17.6	17.6	17.4	18.3	16.6	17.0	16.8	17.0	16.8
Glucose (mmol/L)	13.7	14.0	14.1	13.8	14.4	15.3	15.8	16.6	15.8	15.6
Lactate (mmol/L)	2.7	2.7	2.8	2.6	2.6	2.6	2.7	2.8	2.7	2.9

### Histology

**Figure 4:**
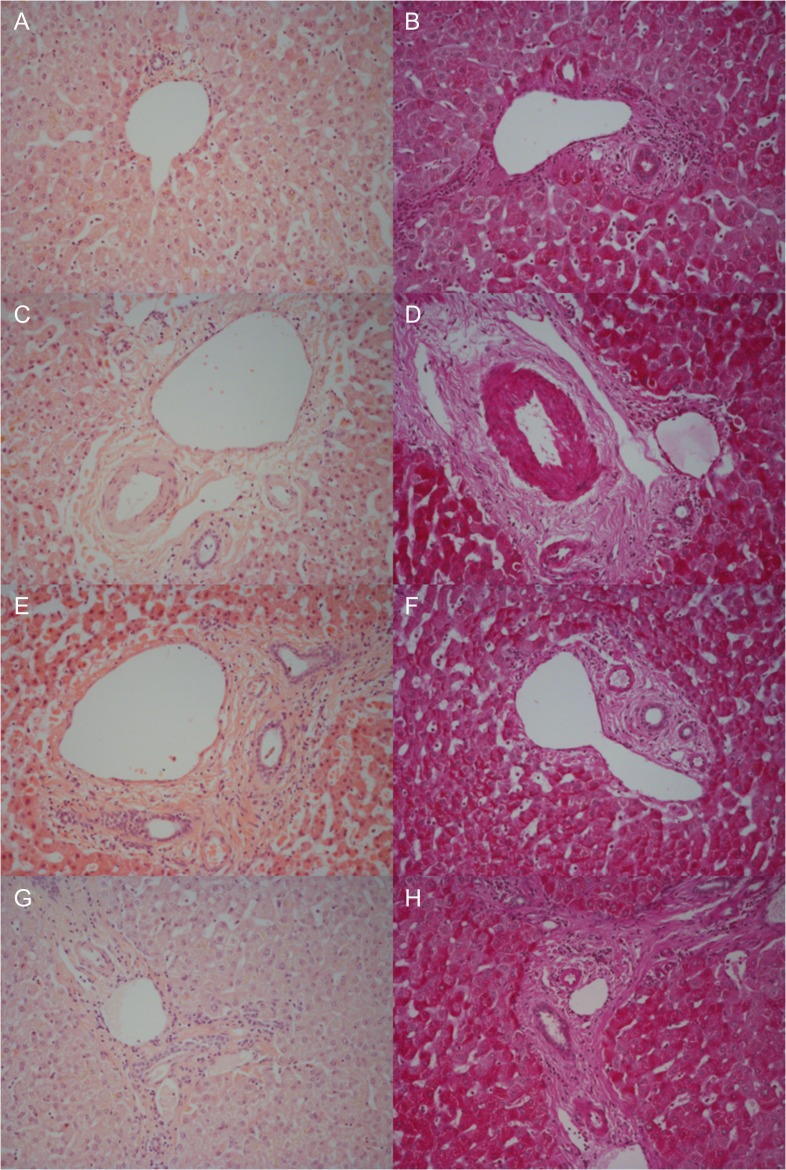
Representative histological samples to assess architectural integrity and necrosis by H&E staining (**A**, **C**, **E**, **G**) as well as glycogen content using Periodic acid-Schiff staining (**B**, **D**, **F**, **H**). Samples taken prior to commencement of NMP-L (A, B), after 3 h of NMP-L (C, D), after 6 h of NMP-L (E, F) and after parenchymal splitting with concurrent NMP-L (G, H).

Moderate sinusoidal vasodilatation was noted throughout although milder post-splitting (Fig. 4A, C, E and G). There were no necrotic hepatocytes post-splitting (Fig. 4G). Macrovesicular steatosis was low (< 10%) pre-NMP-L (Fig. 4A) and remained unchanged (Fig. 4G). There was no intrahepatic bile duct injury. The trend was for increasing PAS staining over time (Fig. 4B, D, F and H).

## DISCUSSION

This is the first study demonstrating that splitting a liver with concurrent NMP-L throughout is feasible and viability is maintained. Furthermore, this has been achieved in a liver unsuitable for transplantation. No studies to date have investigated liver splitting with concurrent NMP-L throughout splitting using either normothermic, subnormothermic or hypothermic strategies.

This technique presents advantages over existing methods. There is no increasing temperature and rewarming under ischaemic conditions seen with cold *ex situ* splitting. Whilst possible during *in situ* splitting, concurrent NMP-L provides *ex situ* direct visualization of perfused segments. Throughout splitting the cut surface can be inspected and vessels ligated to ensure haemostasis. Continuation of NMP-L permits continuous viability assessment. This may contribute to recipient selection, aid informed decision-making and facilitate logistics of transplanting two grafts by preventing long CIT. We have shown that *ex situ* splitting is feasible in ECD livers performed in combination with NMP-L, and results in pre-transplant biochemical, histological and Doppler findings that are compatible with transplantation.

Liver splitting during NMP-L has been described using the OrganOx® *metra™* device. Due to the closed circuit, a hanging procedure was implemented to facilitate dissection whilst minimizing fluid loss from the cut surface. Here perfusion was paused for 6 min to separate the grafts and yielded only an extended right segment still perfused [5]. Specific advantages of an open circuit include: superior manipulation; reduced risk of compromised flow; potential to perform and assess integrity of vascular reconstruction: the ability to perform the complete splitting procedure with concurrent NMP-L.

*Ex situ* splitting with NMP-L allows exploration of splitting ECD livers and may increase the donor pool enabling two recipients to be transplanted from a single donor, it could also facilitate training in liver splitting and provide controls for research.

Classical left lateral + right trisegmentectomy liver splitting with concurrent NMP-L is feasible, maintaining viability of both lobes, combining ‘normal’ physiological conditions and reduced CIT with the ease of *ex situ* splitting, without impacting on organ retrieval. Future work will allow improved monitoring and facilitate logistics.

## Supplementary Material

Supplementary Data
